# Microcarrier-Based Culture of Human Pluripotent Stem-Cell-Derived Retinal Pigmented Epithelium

**DOI:** 10.3390/bioengineering9070297

**Published:** 2022-07-04

**Authors:** Mohamed A. Faynus, Jeffrey K. Bailey, Britney O. Pennington, Mika Katsura, Duncan A. Proctor, Ashley K. Yeh, Sneha Menon, Dylan G. Choi, Jane S. Lebkowski, Lincoln V. Johnson, Dennis O. Clegg

**Affiliations:** 1 Program for Biomolecular Science and Engineering, University of California, Santa Barbara, CA 93106, USA; 2 Center for Stem Cell Biology and Engineering, Neuroscience Research Institute, University of California, Santa Barbara, CA 93106, USA; jkbailey@ucsb.edu (J.K.B.); bop@ucsb.edu (B.O.P.); mikakatsura@ucsb.edu (M.K.); duncan@ucsb.edu (D.A.P.); ashleyyeh@umail.ucsb.edu (A.K.Y.); snehamenon@ucsb.edu (S.M.); dylanchoi@ucsb.edu (D.G.C.); clegg@ucsb.edu (D.O.C.); 3 Department of Molecular, Cellular and Developmental Biology, University of California, Santa Barbara, CA 93106, USA; 4 College of Creative Studies, Chemistry and Biochemistry, University of California, Santa Barbara, CA 93106, USA; 5 Regenerative Patch Technologies LLC, Portola Valley, CA 94028, USA; jane@regenerativepatch.com (J.S.L.); linc@regenerativepatch.com (L.V.J.); 6 Program in Biological Engineering, University of California, Santa Barbara, CA 93106, USA

**Keywords:** microcarriers, stem cells, retinal pigment epithelium

## Abstract

Dry age-related macular degeneration (AMD) is estimated to impact nearly 300 million individuals globally by 2040. While no treatment options are currently available, multiple clinical trials investigating retinal pigmented epithelial cells derived from human pluripotent stem cells (hPSC-RPE) as a cellular replacement therapeutic are currently underway. It has been estimated that a production capacity of >10^9^ RPE cells annually would be required to treat the afflicted population, but current manufacturing protocols are limited, being labor-intensive and time-consuming. Microcarrier technology has enabled high-density propagation of many adherent mammalian cell types via monolayer culture on surfaces of uM-diameter matrix spheres; however, few studies have explored microcarrier-based culture of RPE cells. Here, we provide an approach to the growth, maturation, and differentiation of hPSC-RPE cells on Cytodex 1 (C1) and Cytodex 3 (C3) microcarriers. We demonstrate that hPSC-RPE cells adhere to microcarriers coated with Matrigel, vitronectin or collagen, and mature in vitro to exhibit characteristic epithelial cell morphology and pigmentation. Microcarrier-grown hPSC-RPE cells (mcRPE) are viable; metabolically active; express RPE signature genes including *BEST1*, *RPE65*, *TYRP1*, and *PMEL17*; secrete the trophic factors PEDF and VEGF; and demonstrate phagocytosis of photoreceptor outer segments. Furthermore, we show that undifferentiated hESCs also adhere to Matrigel-coated microcarriers and are amenable to directed RPE differentiation. The capacity to support hPSC-RPE cell cultures using microcarriers enables efficient large-scale production of therapeutic RPE cells sufficient to meet the treatment demands of a large AMD patient population.

## 1. Introduction

Age-related macular degeneration (AMD) is the primary cause of blindness in people over 65 years of age. It has been estimated that in 2020, AMD affected 196 million people worldwide, a number that is projected to increase to 288 million by 2040 [1]. Genetic factors and smoking have been linked to an increased risk of AMD. A hallmark of the disease is dysfunction of the retinal pigmented epithelium (RPE), the monolayer of cells overlying the retina, that results secondarily in a loss of fine acuity in the central field of vision [2,3,4,5]. To date, there are no approved therapies available to treat the most common form of AMD, non-exudative “dry” geographic atrophy, which affects approximately 90% of the patient population [6].

Human pluripotent stem cells (hPSCs) have the potential to generate every cell type, including RPE cells, and hPSC-derived RPE (hPSC-RPE) cells are currently being tested in clinical trials as therapeutics for non-exudative AMD [7,8]. Results from Phase I clinical trials demonstrate that hPSC-RPE cells administered either as a suspension or as a monolayer of adherent cells supported by a scaffold – stabilizes or improves best-corrected visual acuity, and also restores fixation and native retinal structure in some patients when compared to the untreated eye [9,10,11]. 

RPE cells also secrete bioactive factors that exhibit the potential for pharmaceutical applications. Pigment epithelium-derived factor (PEDF) has been reported to have neurotrophic, neuroprotective, and anti-cancer properties, and vascular endothelial growth factor (VEGF) has been shown to exhibit neuroprotective effects in in vitro models of ischemia and animal models of Parkinson’s disease [12,13,14,15]. Therefore, hPSC-RPE cells have the potential to be used directly as a cellular therapy or serve as a source of secreted biologics. 

However, the lack of technology for the manufacture of sufficient quantities of RPE cells to meet the therapeutic demands has been recognized as an obstacle to the widespread use of RPE-based cellular therapies [16,17]. Approaches to large-scale mammalian cell culture include roller bottles and multilayer flasks, both of which are cumbersome to manipulate, require specialized equipment, and require inordinate space in manufacturing facilities [18]. A more promising technology for high-capacity adherent cell culture is a bioreactor-based suspension culture of microcarrier, micrometer-sized, spherical support matrices that provide relatively large surface areas in small volumes. When compared to 2D tissue culture options, microcarriers provide for a wider range of possible cell yields with the lowest cost of goods [18,19,20].

A variety of microcarriers are commercially available, with variations in composition, size, porosity, stiffness, and biochemical surface functionalization [19]. Cytodex 1 (C1) and Cytodex 3 (C3) are designed to support culture of a wide range of animal cell types, are comprised of biologically inert, cross-linked dextran matrices, and provide surface areas of 4400 cm^2^/g and 2700 cm^2^/g, respectively [21]. C1 carries a positive charge, and C3 is functionalized with denatured type 1 collagen derived from porcine skin. The latter is reported to support cells that have difficulty adhering to tissue culture plastic [22]. However, it has yet to be determined whether these microcarriers are applicable to the culture of hPSC-RPE cells.

Previous studies have shown that RPE cells isolated from both fetal and human postmortem eyes can attach to microcarriers, proliferate, form pigment, and secrete PEDF and VEGF [23,24,25]. However, human eye tissues lack the capacity to generate quantities of RPE cells sufficient for widespread therapeutic use. Since hPSCs have the potential to generate a theoretically unlimited number of RPE cells, they are a promising option to use in combination with the high-capacity culture technology of microcarriers in order to meet the clinical demand. 

Here, we demonstrate that hPSC-RPE cultured on Cytodex 1 microcarriers coated with either Matrigel (C1Mg) or vitronectin (C1Vn), as well as Cytodex 3 (C3Clg) microcarriers express the mature RPE phenotype as well as RPE secretory and phagocytic functions. We also demonstrate that microcarrier-associated RPE (mcRPE) may be harvested using an xeno-free dissociation reagent. Furthermore, we show that hESCs may be directly differentiated into progenitor RPE cells in 14 days on C1Mg microcarriers. Interestingly, we found that C1Vn microcarriers exhibited the highest coverage of RPE cells and elevated levels of VEGF and PEDF after 30 days of culture compared to Matrigel and collagen-coated counterparts. These results provide proof-of-concept for large-scale culture of mature, functional stem-cell-derived RPE for potential application as cell-based therapies. 

## 2. Materials and Methods

### 2.1. Microcarrier Preparation 

To yield 750 cm^2^ of microcarrier surface area, 250 mg of blank C1 and 375 mg of C3 were resuspended in 12.5 mL of phosphate-buffered saline with calcium and magnesium (PBS+/+) and swelled for 5 h with gentle agitation at room temperature to hydrate and expand beads to maximal surface area. The swelled microcarrier suspensions were sterilized in glass containers at 115 °C and 15 PSI for 15 mins and cooled at room temperature for 1 h, transferred to fresh 50 mL conical tubes and stored at 4 °C.

### 2.2. Microcarrier Coating and Seeding

To yield required C1, a volume equating to 3.8 cm^2^ surface area was calculated from the initial 750 cm^2^ stock; this volume was transferred to fresh 15 mL conical tubes, resuspended in 12.5 mL CTS AIM-V (Gibco #A3830801) medium and incubated for 1 h at 37 °C to allow proper reswelling. Swelled C1 was subsequently coated with hESC-qualified Matrigel (Corning (Corning, AZ, USA) #354277) or 20ug/mL of CTS human recombinant vitronectin (Thermo Fisher Scientific (Waltham, MA, USA) #A27940) for 1 hr. Samples were manually agitated to resuspend settled C1 approximately every 5 min. A volume equating to 3.8 cm^2^ C3 was similarly equilibrated with CTS AIM-V medium. Frozen stocks of hPSC-RPE were generated from H9 (WA09) human embryonic stem cells (WiCell) by spontaneous differentiation, thawed, cultured, resuspended and seeded at 2 × 10^5^ cells/cm^2^ in all three coating conditions. Microcarrier-seeded RPE cells (mcRPE) were transferred 1 day post seeding (1DPS) to ultra-low attachment 6-well plates (Corning #6906A25) and cultured in CTS AIM-V at 37 °C with 5% CO_2_.

### 2.3. Cell Viability Assay

Samples of mcRPE equal to 0.4 cm^2^ were collected 30DPS. Cell viability was assessed by staining mcRPE with propidium iodide (PI, Life Technologies (Carlsbad, CA, USA), 1:1000) and Hoechst 33342 (Life Technologies, 1:1000) in PBS+/+ for 10 min at 37 °C with 5% CO_2_. To generate a non-viable cell control for propidium iodide staining, mcRPE was incubated with 20 ug/mL digitonin in CTS AIM-V for 24 hrs. Three representative fields of view were collected using a Leica SP8 confocal microscope and analyzed using FIJI ImageJ software. The number of PI-positive, non-viable cells was subtracted from the total cell count (Hoechst) to determine the number of viable cells, and the percent viable was calculated by division using total cell number.

### 2.4. AlamarBlue Metabolic Assay and CytoTox-Fluor Cytotoxicity Assay

Samples of microcarriers equal to 0.4 cm^2^ were collected 30DPS and exposed to alamarBlue High-Sensitivity Cell Viability Reagent (Invitrogen (Waltham, MA, USA) #A50101) diluted 1:10 and CytoTox-Fluor Cytotoxicity Substrate bis-AAF-R110 (Promega (Madison, WI, USA) #G609A) diluted 1:1000 in CTS AIM-V medium for 1 h at 37 °C and 5% CO_2_. Supernatant samples were collected and assayed in technical duplicates for alamarBlue and CytoTox-Fluor fluorescence using a Synergy H1 Hybrid Multi-Mode Microplate Reader (BioTek, Winooski, VT, USA) at Ex/Em wavelengths of 560/590 nm and 485/520 nm, respectively. To calculate fold over blank, the raw relative fluorescence (RF) output for each sample was divided by the fluorescence of a blank control of CTS AIM-V medium supplemented with alamarBlue or CytoTox-Fluor that was co-incubated alongside the test samples. To generate a non-viable cell control, triplicate microcarrier samples were rinsed overnight in CTS AIM-V culture medium and then incubated in culture medium supplemented with 20 µg/mL digitonin (Sigma-Aldrich, St. Louis, MO. USA) at 37 °C and 5% CO_2_. Cytotoxicity and viability were assessed after approximately 5h and 24h of exposure to digitonin, respectively. 

### 2.5. PEDF and VEGF Supernatant ELISA

Cell culture supernatant samples from mcRPE (30DPS) were obtained by incubating volumes of microcarriers containing approximately 0.8 cm^2^ in 48-well plates with 0.5 mL/well of fresh CTS AIM-V medium for 24 h, ±1 h at 37 °C and 5% CO_2_. Brightfield whole-well imaging was performed using a Celigo Image Cytometer (Nexcelom Bioscience, Lawrence, MA, USA), and the number of microcarriers per well was counted using the ImageJ Cell Counter plugin (mean ± s.d., 580 ± 132 microcarriers/well). Samples were frozen at −80 °C until the time of processing. Sandwich ELISAs for Pigment Epithelium-Derived Factor (PEDF) and Vascular Endothelial Growth Factor A (VEGF) were performed using commercially available kits according to the manufacturer’s instructions (PEDF: Abcam (Cambridge, UK) #ab246535, VEGF: Abcam #ab222510). Sample dilutions of 80-fold for the PEDF ELISA and 5-fold for the VEGF ELISA were performed to ensure that all results remained in the linear detection range for each assay. Surface area normalization was based on a mean surface area of 0.091 mm^2^ per microcarrier for Cytodex 1 and 0.071 mm^2^ per microcarrier for Cytodex 3 based on diameters of 170 µm and 150 µm, respectively, with spherical surface area $A=4\pi r^{2}$ [21].

### 2.6. mcRPE Photoreceptor Outersegment Phagocytosis

Purification and labeling of bovine photoreceptor outer segments (POS) was performed as described previously [26]. FITC-labeled POS were incubated with mcRPE at a ratio of 20 POS per cell and mouse IgG1 for 16 h at 37 °C and 5% CO_2_. Microcarriers were subsequently washed five times with PBS+/+ and fixed using 4% paraformaldehyde. For function-blocking experiments, mcRPE were pre-incubated for 1 h with 30 ug/mL α_v_β_5_ integrin antibody (Abcam, #ab177004) followed by a 16 h co-incubation of mcRPE with α_v_β_5_ integrin antibody and FITC-labeled POS. Samples were rinsed, fixed and co-stained with phalloidin (Thermo Fisher Scientific #A22287) to label F-actin and Hoechst to label nuclei. Imaging was performed using a Leica SP8 Resonant Scanning Confocal microscope. POS particles and nuclei were thresholded and 3 representative field over-views were counted using Analyze Particles function with size restrictions; quantification was performed using FIJI ImageJ. 

### 2.7. Reverse Transcription Quantitative Polymerase Chain Reaction (RT-qPCR)

Lysates for RNA purification were produced by triturating mcRPE in RLT lysis buffer (Qiagen (Germantown, MD, USA) #74034). RLT lysates were stored at −80℃, thawed, and homogenized using Qiashredder columns (Qiagen). RNA was purified with the RNeasy kit (Qiagen #74034) and treated with RNase-free DNase to conduct on-column digestion of genomic DNA. Reverse transcription and PCR were performed using the Applied Biosystems AG-pathID One Step PCR kit (Thermo Fisher Scientific #4387391) using TaqMan primer–probe sets (all Thermo Fisher Scientific) for EIF2B2 (Hs00204540_m1), SERF2 (Hs00428481_m1), UBE2R2 (Hs00215107_m1), RPE65 (Hs01071462_m1), TYRP1 (Hs00167051_m1), S100A4 (Hs00243202_m1), PMEL17 (Hs01124465_m1), BEST1 (Hs00188249_m1), OCT4 (Hs00999632_g1), RLBP1 (Hs00165632_m1), RAX (Hs00429459_m1), LHX (Hs00180351_m1) and TYR (Hs00165976_m1). Gene expression was quantified using BioRad (Hercules, CA, USA) CFX Real-Time PCR detection software and expression was normalized as fold-over the geometric mean of the housekeeping genes (EIF2B2, SERF2, UBE2R2). 

### 2.8. Immunocytochemistry 

Microcarriers were collected at 30DPS, blocked and permeabilized with 1% bovine serum albumin and 0.1% Triton X-100 diluted in PBS with calcium and magnesium for 1 h at room temperature. Primary antibody incubations were performed overnight at 4 °C using 1:350 dilutions of mouse anti-PMEL17 (Agilent (Santa Clara, CA, USA) # M0634), mouse anti-BEST1 (Thermo Fisher Scientific # MA116739), mouse anti-RPE65 (Millipore (Burlington, MA, USA) # MAB5428) and rabbit anti-ZO-1 (Thermo Fisher Scientific #40-2200) in block solution (1% BSA PBS). Microcarriers were rinsed thrice with PBS and incubated for 1 h at room temperature with 1:250 dilutions of secondary antibodies: AlexaFluor 633 goat anti-mouse (Life Technologies # A21050) or AlexaFluor 488 donkey anti-rabbit (Invitrogen (Waltham, MA, USA) # A21206) in block solutions. mcRPE were co-stained with 1:1000 Hoechst and phalloidin diluted in PBS for 10 min, rinsed, and the stained microcarriers were placed onto coverslips for imaging using a Leica SP8 Resonant Scanning Confocal microscope. 

### 2.9. hPSC Microcarrier Differentiation

Passage 49 H9 stem cells were thawed and cultured in mTeSR1 medium (Stemcell Technologies (Vancouver, BC, Canada) #85850) in 6-well plates coated with hESC-qualified Matrigel. The cells were passaged when 80% confluent using TrypLE (Thermo Fisher Scientific #12563011) and seeded onto Matrigel-coated Cytodex 1 microcarriers in mTeSR1 containing 10 µM rock inhibitor (Tocris (Bristol, UK) #1254) at a density of 4 × 10^5^ cell/cm^2^ yielding H9_C1Mg. At 2DPS, H9_C1Mg were subjected to RPE differentiation as previously described [27,28]. Briefly, Retinal Differentiation Medium (RDM) was composed of DMEM/F12 (Thermo Fisher Scientific #10565042), 1X B27 (Thermo Fisher Scientific #17504044), 1X N2 (Thermo Fisher Scientific #17502048) and 1X non-essential amino acids (NEAA)(Cytiva (Marlborough, MA, USA) #SH30238.01). From days 0 to day 2, H9_C1Mg was cultured in RDM supplemented with 10 mM nicotinamide (Sigma-Aldrich #N0636), 50 ng/mL Noggin (Fisher Scientific (Hampton, NH, USA) #1967NG025), 10 ng/mL Dickkopf-related Protein-1 (Dkk1) (Fisher Scientific #5439DK010) and 10 ng/mL insulin-like growth factor-1 (IGF-1) (Fisher Scientific #291G1200). From day 2 to day 4, H9_C1Mg was cultured in RDM supplemented with 10 mM nicotinamide, 10 ng/mL Noggin, 10 ng/mL Dkk1 and 10 ng/mL IGF-1. From day 4 to day 6, H9_C1Mg was cultured in RDM supplemented with 100 ng/mL activin A (AA) (PeproTech (Cranbury, NJ, USA) #120-14E), 10 ng/mL Dkk1 and 10 ng/mL IGF-1. From day 6 to day 8, H9_C1Mg was cultured in RDM supplemented with 100 ng/mL AA and 10 uM SU5402 (SantaCruz Biotechnology (Dallas, TX, USA) # sc-204308). From day 8 to day 14, H9_C1Mg was cultured in RDM supplemented with 100 ng/mL AA, 10 uM SU5402 and 3 uM CHIR99021 (Reprocell (Beltsville, MD, USA) #04-0004-02). Samples were harvested on day 14 for RT-qPCR analysis.

### 2.10. Statistical Analysis and Data Display

Data analysis, graphing, and statistics were performed with GraphPad Prism. All data are represented as means with error bars indicating standard error of the mean (SEM). Statistical analysis was performed using paired Student’s t-tests, one-way or two-way ANOVAs with correction for multiple comparisons as appropriate. Significance was assessed using α = 0.05 for all analyses.

## 3. Results

### 3.1. hESC-RPE Attach and Mature on Microcarriers Coated with Extracellular Matrix Proteins

**Figure 1:**
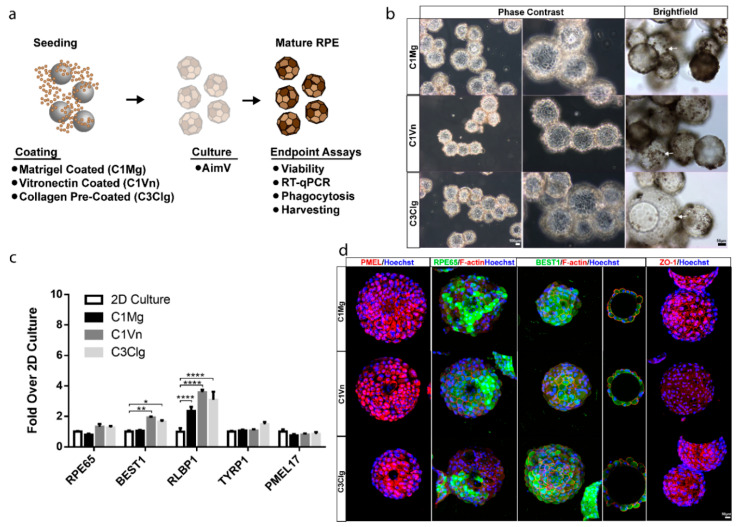
Microcarrier-seeded RPE (mcRPE) mature and express RPE markers (**a**) Outline of experimental approach. Cytodex 1 microcarriers were coated with either Matrigel (C1Mg) or human recombinant vitronectin (C1Vn) and Cytodex 3 microcarriers precoated with denatured porcine-skin collagen (C3Clg) were seeded with hPSC-RPE cells and cultured for 4 weeks before analysis. (**b**) mcRPE mature to develop phase-bright borders, form polygonal morphology and produce pigment (brightfield images, white arrow). (**c**) Expression of RPE marker genes *RPE65*, *BEST1*, *RLBP1*, *TYRP1* and *PMEL17* as detected by RT-qPCR. mcRPE demonstrate similar gene expression profiles compared to 2D controls except for significant differences in C1Vn and C3Clg BEST1 (* *p* = 0.0309 and ** *p* = 0.0012) and C1Mg, C1Vn and C3Clg RLBP1 (**** *p* < 0.0001) (**d**) mcRPE express immunocytochemically detectable markers of RPE maturity and polarization including: premelanosome (PMEL17), retinal pigment epithelium-specific 65 kDa (RPE65), bestrophin-1 (BEST1), zonular occludens-1 (ZO-1) and co-stain with F-actin. Statistical analysis, two-way ANOVA, Sidak’s correction. Data represented as means normalized to 2D controls with error bars indicating standard error of means.

hPSC-RPE cells were seeded onto C1Mg, C1Vn and C3Clg microcarriers and cultured for 4 weeks before analysis (Figure 1a). Microcarrier-cultured hPSC-RPE cells (mcRPE) mature to form an epithelial-like monolayer, display polygonal morphology, exhibit phase-bright borders and undergo pigmentation on all three microcarrier types (Figure 1b, Supplemental Figure S1a). mcRPE identity was assessed by immunocytochemistry (ICC) and RT-qPCR for RPE signature markers. RT-qPCR analyses demonstrated no significant differences in expression of *RPE65*, *TYRP1* and *PMEL17* genes (*p* > 0.05) compared to 2D hPSC-RPE cells cultured on Matrigel. However, mcRPE on C1Vn and C3Clg microcarriers express significantly more *BEST1* (*p* = 0.0309 and *p* = 0.0012, respectively) and mcRPE on C1Mg, C1Vn and C3Clg microcarriers express significantly more *RLBP1* (all three conditions, *p* < 0.0001) compared to 2D controls (Figure 1c). The increased expression of *BEST1* and *RLBP1* may indicate the rapid maturation of mcRPE. mcRPE also expresses PMEL17 and RPE65 proteins as determined by ICC (Figure 1d). Polarization of the mcRPE was evidenced by basolateral localization of BEST1, and membrane-associated accumulations of F-actin and ZO-1 suggested the elaboration of junctional complexes (Figure 1d). 

### 3.2. mcRPE areHighly Viable and Metabolically Active

**Figure 2:**
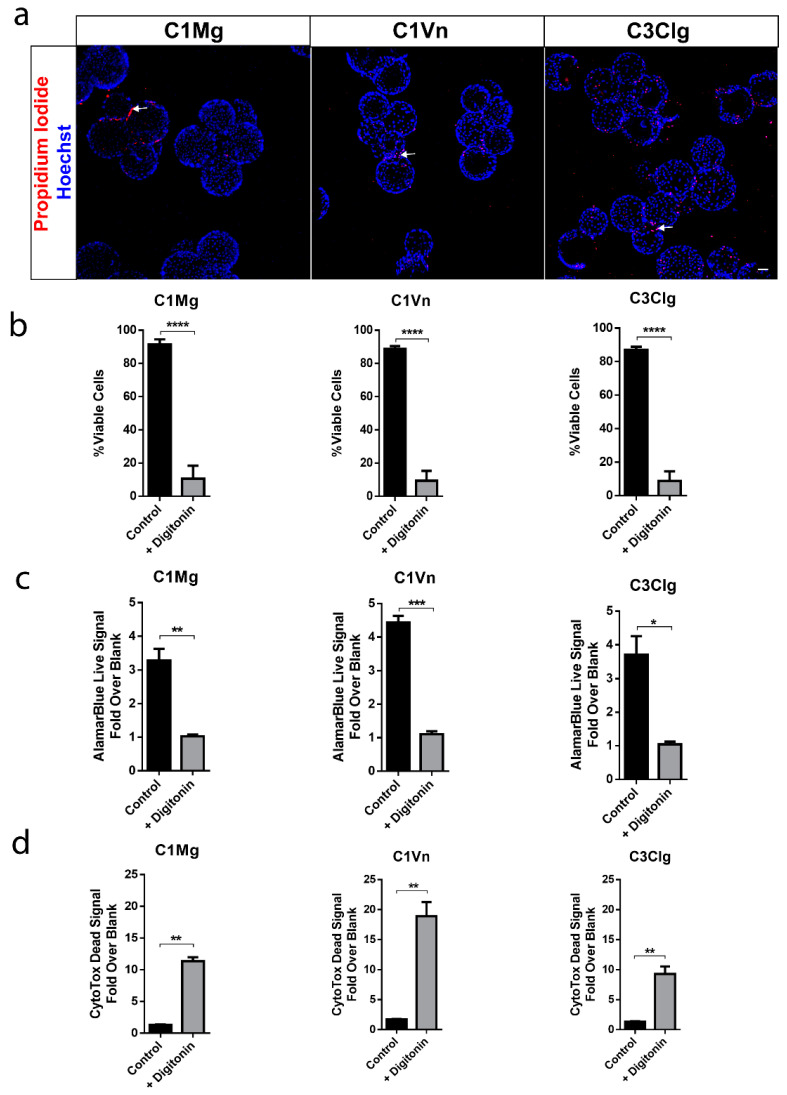
mcRPE are viable and metabolically active. (**a**) mcRPE exhibits high viability on all three microcarrier types with few non-viable cells (red) as demonstrated by propidium iodide nuclear staining. (**b**) Quantification of the propidium iodide staining data revealed an average >88% cell viability for all three microcarriers with a significant decrease when exposed to Digitonin (**** *p* < 0.0001, two-way ANOVA, Sidak’s correction). (**c**) mcRPE demonstrates active metabolism with a decrease when exposed to Digitonin as measured through alamarBlue reagent (** *p* = 0.006, *** *p* = 0.001, * *p* = 0.0105, paired, two-tailed *t* test. (**d**) Cells’ viability was further measured using CytoTox-Fluor assay for dead-cell protease activity. All three microcarrier conditions exhibit minimal protease activity with a significant increase when exposed to digitonin (** *p* < 0.05, paired, two-tailed *t* test). Scale bar 50 µm, data represented as means with error bars indicating standard error of mean.

At four weeks post seeding, the viability of mcRPE was assessed by propidium iodide (PI) staining. Confocal Z projections showed relatively few non-viable, PI-positive nuclei associated with all three microcarrier types (Figure 2a); 88%, 91% and 87% cell viabilities were determined for C1Mg, C1Vn and C3Clg, respectively (each *p* < 0.0001 compared to digitonin control) (Figure 2b). The metabolic activity of mcRPE analyzed using alamarBlue showed average fold-over-blank metabolism ratios of 3.3× for C1Mg, 4.4× for C1Vn and 3.7× for C3Clg. Metabolic activity significantly decreased when mcRPE was permeabilized with digitonin (Figure 2c, C1Mg: *p* = 0.006, C1Vn: *p* = 0.001, C3Clg: *p* = 0.0105). Baseline dead cell protease activity for control mcRPE was 1.3-, 1.7- and 1.3-fold over background for C1Mg, C1Vn and C3Clg, respectively, which significantly increased when mcRPE was incubated with digitonin (Figure 2d, *p* < 0.05). 

### 3.3. mcRPE Can Be Harvested from Microcarriers Using Xeno-Free Methods

To convert large-scale production batches of mcRPE into a clinically relevant form, mcRPE cells must be separated from the microcarrier substrates. Accordingly, we assessed whether 30DPS mcRPE could be harvested from microcarriers using a xeno-free dissociation reagent. The microcarriers were collected and exposed to the TrypLE enzyme for a period of 7 min before initiating harvesting to determine viability as well as cell recovery. After 7 min of TrypLE treatment, all three microcarrier types demonstrated clear indications of cell detachment (Supplemental Figure S2a). RPE suspensions harvested from C1Mg, C1Vn and C3Clg mcRPE cultures after 10 min exposure to TrypLE exhibited 93%, 98% and 91% cell viability, respectively, with C1Vn being significantly more viable than its counterparts (Supplemental Figure S2b, *p* < 0.05). Cell recovery matched this trend with C1Vn yielding significantly more RPE cells (*p* < 0.0001) than C1Mg and C1Vn with C1Mg yielding the fewest recovered cells (Supplemental Figure S2c). These results indicate that mcRPE can be cultured for extended periods and harvested while retaining high viability.

### 3.4. mcRPE Secretes Increasing Amounts of PEDF and VEGF

**Figure 3:**
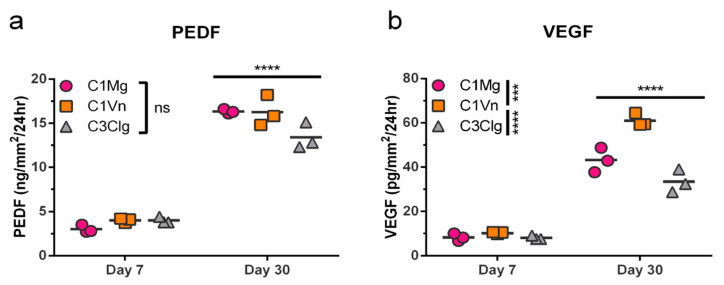
mcRPE secretion of PEDF and VEGF increases between days 7 and 30 post seeding. (**a**) PEDF secretion by mcRPE after 7DPS and 30DPS was quantified by sandwich ELISA for three microcarrier types. mcRPE secreted more PEDF at 30DPS compared to 7DPS, but no significant differences among microcarrier types were observed (ns, not significant). (**b**) VEGF secretion by mcRPE was measured in the same samples by sandwich ELISA. Day 30 mcRPE also secreted more VEGF compared to day 7, and mcRPE on C1Vn secreted higher levels of VEGF than those on C1Mg or C3Clg (*** *p* < 0.001, **** *p* < 0.000, respectively; two-way ANOVA, Sidak’s correction). Horizontal bars indicate mean of three biological replicates (2 × 10^5^ cells per replicate).

The RPE secrete a host of trophic factors necessary to maintain the health and function of the surrounding retina and choroid; two such factors are PEDF and VEGF [14,29]. We assessed the secretion of PEDF and VEGF in medium following 24 hrs of culture with C1Mg, C1Vn or C3Clg mcRPE. Sandwich ELISAs were performed on samples collected at 7DPS and 30DPS. There were no significant differences (ns, *p* > 0.05) in secreted PEDF quantities among the three conditions (Figure 3a); however, significantly more (*p* < 0.0001) PEDF was secreted at day 30 than at day 7 for all three microcarriers, suggesting increasing maturation over time. mcRPE on all three microcarriers also secreted significantly more VEGF at day 30 compared to day 7 (*p* < 0.0001); however, C1Vn mcRPE secreted significantly more than C1Mg and C3Clg at day 30 (*p* < 0.001 and *p* < 0.0001, respectively). The ability of mcRPE cultures to mature and secrete trophic factors supports the concept that future therapeutic applications utilizing the RPE secretome are technically feasible using microcarriers.

### 3.5. mcRPE Phagocytose Photoreceptor Outer Segments

**Figure 4:**
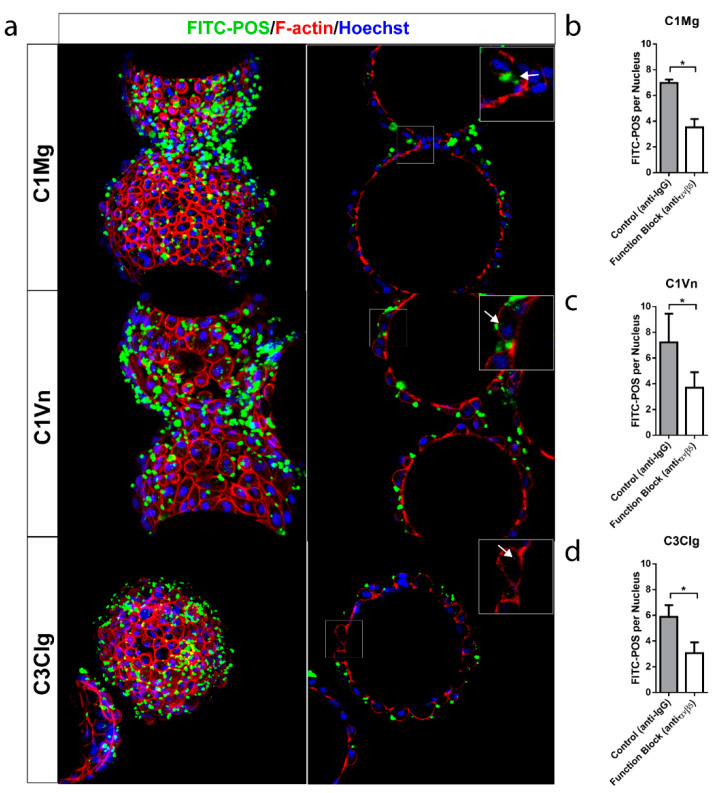
mcRPE demonstrate phagocytic activity (**a**) mcRPEs were exposed to FITC-conjugated bovine POS at a ratio of 20 POS per cell for 16 hrs in the presence of IgG controls or in some instances anti-α_v_β_5_ antibodies before washing and fixation. Confocal Z projections were collected and bound and internalized POS were visualized by FITC (green) and F-actin (red) fluorescence. Single Z slice images demonstrate internal particulates (inset, white arrow) indicating engulfment. (**b**–**d**) Representative field of view confocal Z projections (*n* = 3 FOV, *n* = 150 nuclei per FOV) were collected for both control IgG and function-blocking conditions and number of FITC-POS per cell (based on Hoechst staining) was assessed. All mcRPE phagocytose FITC-POS mediated in part through RPE-specific α_v_β_5_ integrin receptors as shown by the significant decrease in FITC-POS per cell when exposed to function-blocking antibody (* *p* < 0.05, two-way ANOVA, Sidak’s correction). Data presented as means with error bars indicating standard error of means. Scale bars 50 µm, inset 5 µm.

A primary function of the RPE is the daily phagocytosis of photoreceptor outer segments (POS) and the consequent clearing of the subretinal space [30,31,32]. The phagocytic function of mcRPE was assessed by coincubation of mcRPE and fluorescein isothiocyanate (FITC)-conjugated POS (FITC-POS) and visualization of bound and internalized FITC-POS by confocal microscopy. Bound and internalized FITC-POS was observed by relative localization to the cell membrane, labeled by F-actin (Figure 4a). For each condition, the number of FITC-POS foci per nucleus significantly decreased (*p* < 0.05) when mcRPE were co-incubated with function-blocking anti-α_v_β_5_ antibody (Figure 4b–d). mcRPE phagocytosed an average of 7.0 POS for C1Mg, 7.2 for C1Vn and 5.9 for C3Clg; these values significantly decreased (*p* < 0.05) when co-incubated with function-blocking anti-α_v_β_5_ antibody (Figure 4b–d). These results document the phagocytic function of mcRPE.

### 3.6. hESCs Can Be Differentiated into RPE-Progenitors on Microcarriers

**Figure 5:**
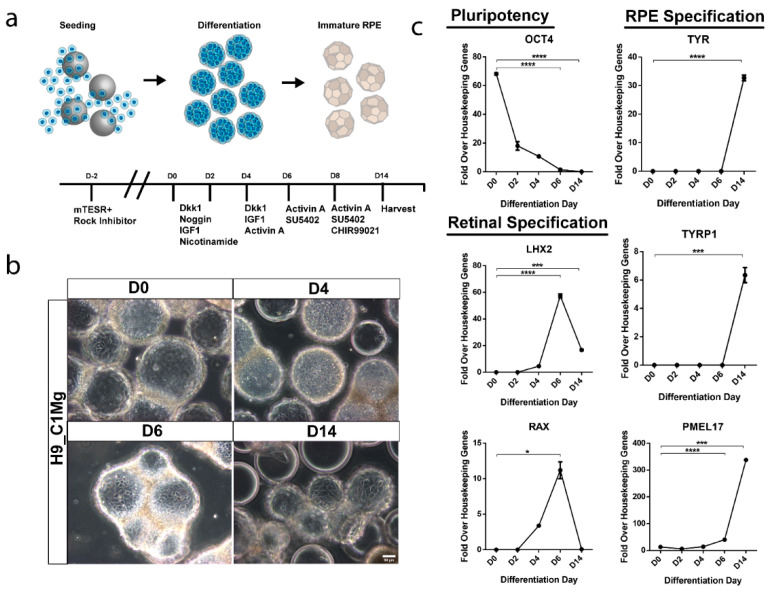
Microcarrier-based differentiation of RPE from hESC. (**a**) Human embryonic stem cells (H9) were seeded on Matrigel-coated microcarriers and differentiated towards an RPE fate using a cocktail of growth factors and small molecules. (**b**) Phase contrast microscopy demonstrates retention of cells throughout the differentiation process. (**c**) Differentiation and identity specification was assessed by RT-qPCR. Pluripotency marker OCT4 significantly decreased by 6DPS and 14DPS indicating differentiation (**** *p* < 0.001) while expression of early eye field markers LHX2 and RAX significantly increased by 6DPS and proceeded to decrease by 14DPS suggesting retinal specification and transition towards a terminal cell type (**** *p* < 0.001, *** *p* = 0.001 and * *p* < 0.05). RPE markers tyrosinase (*TYR*), tyrosinase related protein 1 (*TYRP1*) and premelanosome *(PMEL17)* significantly increase by 14DPS suggesting an RPE fate (**** *p* < 0.0001 and *** *p* = 0.001). Statistical analysis two-way ANOVA, Sidak’s correction.

Current manufacturing procedures for RPE cells destined for clinical application typically involve the differentiation of hPSCs in 2D culture [9,11,33]. In order to assess the potential for integration of microcarrier culture in this step, which would facilitate increased scaling at this early manufacturing stage, C1Mg was seeded with H9 stem cells to yield H9_C1Mg. Subsequent directed differentiation toward an RPE fate was performed using growth factors and small molecules according to our previously published protocol for 2D cultures (Figure 5a) [27,28]. The H9 cells readily attached to C1Mg and microcarrier aggregates formed, likely due to the propensity of H9 cells for self-adherence (Figure 5b) [34]. Gene expression profiling showed differentiation using pluripotency, retinal progenitor and RPE markers. The pluripotency marker OCT4 significantly decreased in expression by 6DPS and 14DPS (*p* < 0.0001, respectively, compared to D0 controls) indicating diminishing pluripotency, while early eye field markers, LIM homeobox protein 2 (LHX2) and retina and anterior neural fold homeobox (RAX), significantly increased by 6DPS and decreased by 14DPS (*p* < 0.0001 and *p* < 0.05, respectively), similar to that observed in 2D culture [27,28]. Further quantification of the RPE markers TYR, TYRP1 and PMEL17 revealed significantly increased (*p* < 0.0001, *p* < 0.001 and *p* = 0.001, respectively) expression by 14DPS supporting an early RPE-specific cell fate determination (Figure 5c). These results establish a basis for an integrated approach to the production of clinically relevant quantities of hPSC-RPE for cell-based therapies.

## 4. Discussion

The results described here demonstrate the technical feasibility of microcarrier hPSC-RPE cell culture as a candidate method for the large-scale manufacturing of RPE-based cellular therapeutics. All three microcarrier substrates examined (C1Mg, C1Vn, and C3Clg) yielded mcRPE cultures that exhibited typical RPE characteristics including pigmentation, polygonal morphology, polarization, expression of RPE marker genes, trophic factor secretion, and phagocytic function [7]. While several metrics indicated similar mcRPE properties across the different microcarrier types, C1Vn mcRPE had higher levels of RPE marker gene expression, VEGF secretion, and post-harvest viability, indicating C1Vn as a preferred substrate for further optimization. The increased properties attributed to C1Vn could relate to substrate coating. We observed clumping of Matrigel during the coating procedure and there are studies suggesting complications with C3Clg, both likely impacting downstream cellular adhesion [23]. 

The scale-up of production has been recognized as an important milestone to the commercialization of RPE-cell based therapies, and microcarriers provide distinct advantages compared to static 2D culture systems in terms of available surface area, surface to volume ratio, scalability, accessibility, and cost that may help meet patient demand [16]. The number of patients with geographic atrophy (GA) in the United States alone is projected to be 1.2 million in 2033 with an annual incidence of 160,000 [35]. The clinical dose for proposed RPE-based cellular therapies ranges between 50,000 to 150,000 cells administered either as an injection of a cellular suspension or as an implantation of scaffold-supported cells [7]. Assuming only half of the new GA patients in the United States receives an RPE cell-based therapy, a conservative estimate of the number of cells required annually can be calculated as:

(1) $$\frac{100,000\;RPE\;cells}{clinical\;dose}\times \frac{1\;dose}{patient}\times \frac{80,000\;patients}{year}=\frac{8\times 10^{9}RPE\;cells}{year}$$

This estimate assumes that only a single dose of cells will be needed per patient, while in practice it is likely that additional doses will be shipped to the clinical site for redundancy in case of complications or to treat both eyes when necessary. Additionally, this estimate does not account for the number of RPE cells required for quality control assays and process validation. The amount of surface area that is needed to supply 8 × 10^9^ RPE cells/year is approximately 3174 cm^2^/month, which can be achieved by the monthly use of either forty-two T-75 flasks, fourteen T-225 flasks, or by using microcarriers in a bioreactor volume of less than one liter (Supplemental Table S1). This demonstrates that microcarriers offer a substantial advantage over 2D-culture methods and can provide considerable savings in manufacturing space and cost of goods.

It has been recognized that production of cellular therapies must ultimately move toward automated manufacturing lines consisting of closed, modular, and scalable components. Microcarriers are especially compatible with each of these requirements [20]. For example, in a closed production line, an operator would initiate a batch by inoculating microcarriers with a starting culture of hPSCs, and all downstream steps would be performed by automated modules that are closed from the environment to prevent contamination while ensuring reproducibility. To this end, studies have reported that hPSCs can be cultured on microcarriers using a xeno-free, serum-free, and chemically defined culture medium, and that stem cells and human neural progenitor cells seeded on microcarriers can differentiate into multiple cell types [17,18,19,36,37]. This report documents the directed differentiation of hESCs into an RPE-cell fate conducted entirely on microcarriers. H9 hESCs differentiated on C1Mg microcarriers exhibit a similar pattern of gene expression to hESCs undergoing the same protocol in a 2D format, including transient RAX and LHX2 expression followed by upregulation of RPE markers such as PMEL17, TYRP1, and TYR (Figure 5a–c) [27,28]. We did observe some cell death during the differentiation process and some adhesion between H9_C1Mg, but this does not pose significant technical complications for downstream harvesting. Finally, xeno-free reagents compatible with cGMP manufacturing can be used to harvest highly viable, mature RPE cells from C1Mg, C1Vn, and C3Clg microcarriers (Supplemental Figure S2a–c). Future studies will be necessary to determine how these individual processes can best be integrated into a single closed and automated production line. 

For large-scale microcarrier-based cell manufacturing and automation, suitable bioreactors will likely be required, and it will be important to demonstrate RPE cell health and functionality when cultured in a bioreactor system. Different bioreactor designs are expected to introduce varying degrees of hydrodynamic shear stress as the microcarriers are maintained in suspension. Vertical-wheel bioreactors are of particular interest, as they have been shown to reduce shear force, support the expansion of human iPSCs, and also improve the viability of human mesenchymal stem cells when compared to stirred-tank bioreactors [17,38]. Whether these systems are appropriate for hPSC-derived mcRPE remains an important issue to be addressed.

Microcarrier cultures have also been used to mass-produce secreted biologics such as vaccines, recombinant proteins, and monoclonal antibodies [22]. Therefore, in addition to providing cells for regenerative therapies, mcRPE could also serve as a source for biopharmaceuticals due to their diverse secretome [39]. In the present study, we demonstrate that secretion of PEDF and VEGF by mcRPE significantly increases between 7 days and 30 days post-seeding as is typical for 2D RPE cultures (Figure 3a,b), and studies have reported the potential applications of these two factors [40]. PEDF has been reported to (i) exhibit neuroprotective effects in Parkinson’s disease as well as in in vitro models of motor neuron degeneration, (ii) confer anti-inflammatory properties in an animal model of diabetic retinopathy, and (iii) exhibit multifaceted anti-cancer and anti-tumor effects [29,41]. Although the dysregulation of VEGF is a hallmark of pathological conditions such as tumor angiogenesis and exudative “wet” AMD, this factor is crucial for normal vasculature physiology and has also been shown to mediate neuroprotection and even neurorescue of dopaminergic neurons in vivo and in vitro models of Parkinson’s Disease [12,15,42]. Therefore, mcRPE may offer a scalable approach for mass-production of RPE-secreted biologics such as PEDF and VEGF.

## 5. Conclusions

RPE replacement therapies are a potentially effective solution in the treatment of retinal diseases and several clinical trials are underway [9,11]. Here, we address one of the current challenges associated with clinical RPE cell production: the scale-up of manufacture. The results described in this report support the further investigation of microcarrier technologies for therapeutic hPSC-RPE cell production.

## Data Availability

Exclude.

## Supplementary Materials

The following supporting information can be downloaded at https://www.mdpi.com/article/10.3390/bioengineering9070297/s1. Figure S1. mcRPE mature and pigment over 30-day culture period. Phase contrast images demonstrate attachment of hESC-RPE cells to Matrigel and recombinant human vitronectin coated Cytodex 1 as well as pre-coated collagen Cyto-dex 3 microcarriers. mcRPE display cobblestone morphology, phase bright borders and pigmentation 29DPS. Figure S2. Harvesting hESC-RPE cells from microcarriers using a xeno-free enzymatic disassociation reagent. (a) mcRPE were collected at a density of 2 × 10^5^ cells per well and exposed to TrypLE enzyme for a period of 7 min. Cells were still attached at 5 min (white arrows) and were completely dissociated after mechanical perturbation at 7 min. Dissoci-ated cells were clearly visualized post perturbation (yellow arrow) and harvested by filtration to separate microcarriers from cells. Empty microcarriers (green arrow) after filtration process demonstrates appropriate harvesting of hESC-RPE cells. (b) Viability of harvested hESC-RPE cells were assessed using Acridine Orange/DAPI exclusion on an NC200. All three conditions demonstrate high viability with C1Vn exhibiting significantly greater viability than C1Mg and C3Clg (** *p* < 0.05). (c) Cell recovery was quantified using Acridine Orange/DAPI and compared to initial starting density equat-ing to an approximate 2 × 10^5^ cells. All three conditions were amenable to cell recovery but C1Vn exhibited a significantly greater percentage of recovered cells compared to C1Mg and C3Clg. C1Mg was the least favorable coating for cell re-covery (**** *p* < 0.001). Statistical analysis, one way ANOVA, Tukey’s correction. Table S1. Calculations to determine the required surface area to meet the estimated patient demand and approaches to achieve.
