# Supplementary material for: Microcarrier-Based Culture of Human Pluripotent Stem-Cell-Derived Retinal Pigmented Epithelium
Source: Bioengineering (Basel). 2022 Jul 4;9(7):297. doi: 10.3390/bioengineering9070297 (PMC9311890; doi:10.3390/bioengineering9070297)
Supplement: Supplementary file 1 [file bioengineering-09-00297-s001.zip › bioengineering-1765216-supplementary.pdf]

Supplementary Information

Microcarrier-Based Culture of Human Pluripotent Stem-Cell-Derived Retinal Pigmented Epithelium

Mohamed A. Faynus [1,2,*], Jeffrey K. Bailey [2], Britney O. Pennington [2], Mika Katsura [2], Duncan A. Proctor [2,3] Ashley K. Yeh [2,3], Sneha Menon [2,3], Dylan G. Choi [2,4], Jane S. Lebkowski [6], Lincoln V. Johnson [6] and Dennis O. Clegg [2,3,5,6]

1 Program for Biomolecular Science and Engineering, University of California, Santa Barbara, CA 93106, USA
2 Center for Stem Cell Biology and Engineering, Neuroscience Research Institute, University of California, Santa Barbara, CA 93106, USA; jkbailey@ucsb.edu (J.K.B.); bop@ucsb.edu (B.O.P.); mikakatsura@ucsb.edu (M.K.); duncan@ucsb.edu (D.A.P.); ashleyyeh@umail.ucsb.edu (A.K.Y.); snehamenon@ucsb.edu (S.M.); dylanchoi@ucsb.edu (D.G.C.); clegg@ucsb.edu (D.O.C.)
3 Department of Molecular, Cellular and Developmental Biology, University of California, Santa Barbara, CA 93106, USA
4 College of Creative Studies, Chemistry and Biochemistry, University of California, Santa Barbara, CA 93106, USA
5 Program in Biological Engineering, University of California, Santa Barbara, CA 93106, USA
6 Regenerative Patch Technologies LLC, Portola Valley, CA 94028, USA; jane@regenerativepatch.com (J.S.L.); linc@regenerativepatch.com (L.V.J.)
* Correspondence: faynus@ucsb.edu

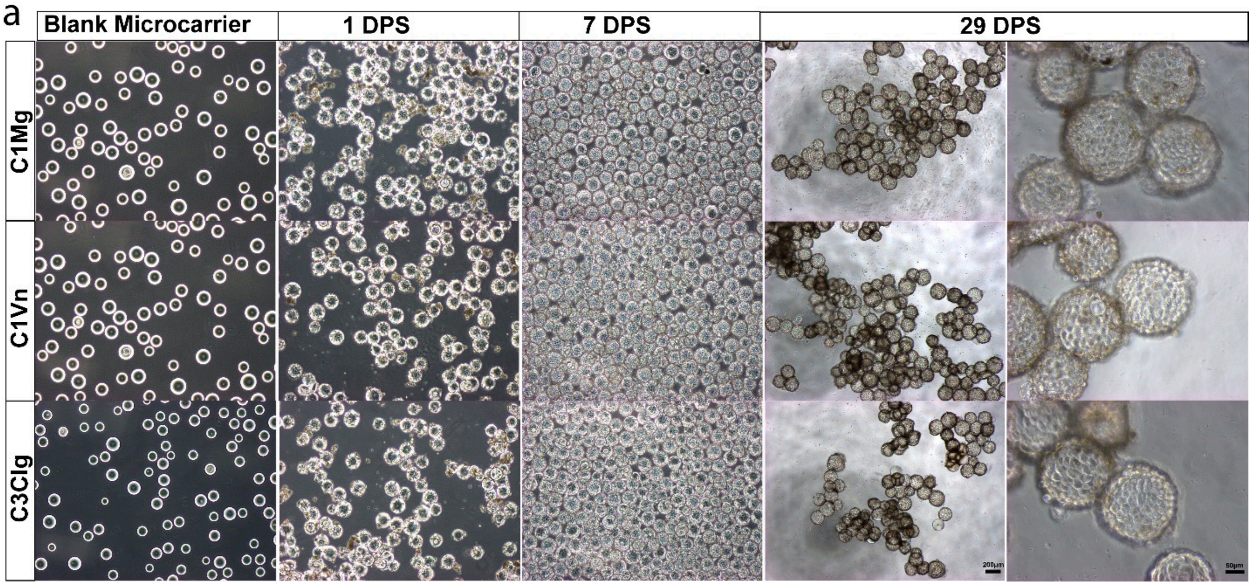

Figure S1. mcRPE mature and pigment over 30-day culture period. Phase contrast images demonstrate attachment of hESC-RPE cells to Matrigel and recombinant human vitronectin coated Cytodex 1 as well as pre-coated collagen Cytodex 3 microcarriers. mcRPE display cobblestone morphology, phase bright borders and pigmentation 29DPS.

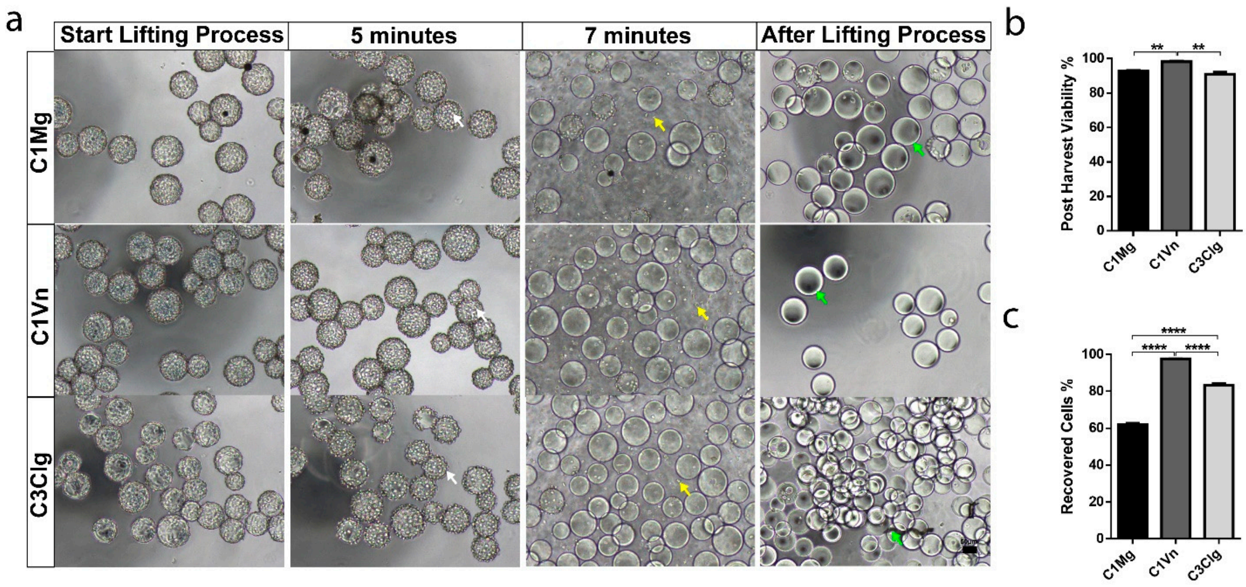

Figure S2. Harvesting hESC-RPE cells from microcarriers using a xeno-free enzymatic disassociation reagent. **a)** mcRPE were collected at a density of $2\text{x}10^5$ cells per well and exposed to TrypLE enzyme for a period of 7 min. Cells were still attached at 5 min (white arrows) and were completely dissociated after mechanical perturbation at 7 minutes. Dissociated cells were clearly visualized post perturbation (yellow arrow) and harvested by filtration to separate microcarriers from cells. Empty microcarriers (green arrow) after filtration process demonstrates appropriate harvesting of hESC-RPE cells. **b)** Viability of harvested hESC-RPE cells were assessed using Acridine Orange/DAPI exclusion on an NC200. All three conditions demonstrate high viability with C1Vn exhibiting significantly greater viability than C1Mg and C3Clg (**$p<0.05$). **c)** Cell recovery was quantified using Acridine Orange/DAPI and compared to initial starting density equating to an approximate $2\text{x}10^5$ cells. All three conditions were amenable to cell recovery but C1Vn exhibited a significantly greater percentage of recovered cells compared to C1Mg and C3Clg. C1Mg was the least favorable coating for cell recovery (****$p<0.001$). Statistical analysis, one way ANOVA, Tukey's correction.

Table S1. Calculations to determine the required surface area to meet the estimated patient demand and approaches to achieve.

*Surface Area Required to Supply 8x10⁹ RPE Cells**	
$\frac{8\times10^9\ RPE\ cells}{year}\times\frac{cm^2}{2.1\times10^5\ RPE\ cells}\times\frac{1\ year}{12\ months}=\frac{3,174cm^2}{month}$	
Approaches to Achieve $\frac{3,174cm^2}{month}$	
**T-75 Flasks**	$\frac{3,174cm^2}{month}\times\frac{1\ flask}{75cm^2}\cong\frac{42\ flasks}{month}$
**T-225 Flasks**	$\frac{3,174cm^2}{month}\times\frac{1\ flask}{225cm^2}\cong\frac{14\ flasks}{month}$
**Cytodex 1**	$\frac{3,174cm^2}{month}\times\frac{g\ of\ Cytodex1}{4400cm^2}\times\frac{L}{5\ g\ of\ Cytodex^{**}}=\frac{144mL\ Bioreactor\ Volume}{month}$
**Cytodex 3**	$\frac{3,174cm^2}{month}\times\frac{g\ of\ Cytodex3}{2700cm^2}\times\frac{L}{5\ g\ of\ Cytodex^{**}}=\frac{235mL\ Bioreactor\ Volume}{month}$

* Estimate of the required cells assumes that only half of the projected number of AMD patients in the United States receives an RPE cell-based therapy [36].

** Microcarrier cultures may contain 1-5g of Cytodex/L [21].
